# Agonistic Anti-CD40 Enhances the CD8^+^ T Cell Response during Vesicular Stomatitis Virus Infection

**DOI:** 10.1371/journal.pone.0106060

**Published:** 2014-08-28

**Authors:** Julianne M. Zickovich, Susan I. Meyer, Hideo Yagita, Joshua J. Obar

**Affiliations:** 1 Department of Microbiology & Immunology, Montana State University, Bozeman, Montana, United States of America; 2 Department of Immunology, Juntendo University, School of Medicine, Toyko, Japan; University of Colorado School of Medicine, United States of America

## Abstract

Intracellular pathogens are capable of inducing vigorous CD8^+^ T cell responses. However, we do not entirely understand the factors driving the generation of large pools of highly protective memory CD8^+^ T cells. Here, we studied the generation of endogenous ovalbumin-specific memory CD8^+^ T cells following infection with recombinant vesicular stomatitis virus (VSV) and *Listeria monocytogenes* (LM). VSV infection resulted in the generation of a large ovalbumin-specific memory CD8^+^ T cell population, which provided minimal protective immunity that waned with time. In contrast, the CD8^+^ T cell population of LM-ova provided protective immunity and remained stable with time. Agonistic CD40 stimulation during CD8^+^ T cell priming in response to VSV infection enabled the resultant memory CD8^+^ T cell population to provide strong protective immunity against secondary infection. Enhanced protective immunity by agonistic anti-CD40 was dependent on CD70. Agonistic anti-CD40 not only enhanced the size of the resultant memory CD8^+^ T cell population, but enhanced their polyfunctionality and sensitivity to antigen. Our data suggest that immunomodulation of CD40 signaling may be a key adjuvant to enhance CD8^+^ T cell response during development of VSV vaccine strategies.

## Introduction

The goal of any vaccine is to provide long-term protective immunity against the target antigen. Effective T cell vaccines are highly desirable for prophylaxis and immunotherapy of chronic infections and tumors [1]. In general, T cell responses can be divided into four distinct phases: activation, expansion, contraction, and memory. The activation of a CD8^+^ T cell response is initiated by peptide:MHC presentation to cognate naïve T cells by professional antigen-presenting cells. After activation, CD8^+^ T cells undergo a rapid expansion whereby they increase in numbers by up to 50,000-fold [2], [3], [4]. Coincidently, activated CD8^+^ T cells undergo a dramatic genetic reprogramming, resulting in expression of their cytotoxic effector program [5]. Activation and genetic reprogramming of naïve CD8^+^ T cells to generate effector and memory T cells requires three types of signals: 1) TCR engagement with cognate antigen presented by MHC, 2) engagement of co-stimulatory molecules, and 3) cytokine signaling [6]. After extensive proliferation and expansion of the pathogen-specific CD8^+^ T cell population, ∼90–95% of the effector CD8^+^ T cells undergo apoptosis, leaving behind the long-lived memory CD8^+^ T cell population [7]. The 5–10% of effector cytotoxic CD8^+^ T cells which survive long-term can be distinguished from the short-lived cytotoxic CD8^+^ T cells based on their expression of CD127 (IL-7Rα) and KLRG1, respectively [8]. The population of long-lived memory CD8^+^ T cells mature with time [5], [9]. Memory CD8^+^ T cells provide enhanced protection from secondary encounter with the pathogen due in part to the rapid re-expression of effector functions and localization to non-lymphoid tissues [10], [11].

Only within the last decade have the exogenous and endogenous signals necessary for the differentiation of effector cytotoxic CD8^+^ T cells and memory-precursor CD8^+^ T cells begun to be elucidated. TCR or cytokine mediated signals alone are not sufficient for KLRG1 expressing [12], suggesting that numerous signals including TCR engagement, cytokine signaling, and signaling with co-stimulatory pathways are involved to provide full CD8^+^ T cell engagement and subsequent memory development. Importantly, the factors regulating the differentiation pathway of effector and memory CD8^+^ T cell populations are dependent on the infectious agent or vaccination protocol utilized [13], [14]. In an overly simplistic view, a highly pro-inflammatory environment (i.e. IL-2, IL-12, IL-27) favors short-lived, terminal effector CD8^+^ T cell differentiation, while anti-inflammatory cues (i.e. IL-10) favor memory CD8^+^ T cell development [15], [16]. To date, numerous methods for the induction of T cell memory have been utilized with mixed success [17], but the functionality and protective ability of resultant memory CD8^+^ T cell populations remain understudied.

One of the best correlates of CD8^+^ T cell mediated protective immunity or control of persistent infections has been induction and maintenance of polyfunctional T cell populations [18], [19]. CD4^+^ T cell help during CD8^+^ T cell priming is important for the induction of highly functional CD8^+^ T cells [20], [21], [22]. In a number of situations, CD4^+^ T cells have been shown to regulate CD8^+^ T cell responses potentially through CD40/CD154 signaling [23], [24], [25], [26]. Additionally, use of agonistic anti-CD40 mAbs during peptide vaccination act synergistically with TLR agonists and other adjuvants in the induction of protective CD8^+^ T cells [27], [28]. CD8^+^ T cell induction by different pathogens vary in their dependency on CD4^+^ T cells and CD40/CD154 signaling [25], [29]. Because of these differences, we sought to address whether a vaccine vector of an immunization protocol influenced the outcome of the CD8^+^ T cell response.

In this study, we found that while vesicular stomatitis virus (VSV) initially induce a protective memory CD8^+^ T cell population, with time the protective ability of the VSV-induced memory CD8^+^ T cell population waned. Provision of CD40 signaling during CD8^+^ T cell priming enhanced the functionality and protective ability of the memory CD8^+^ T cells induced by VSV infection at later time points. CD70 signaling was necessary for enhancing memory CD8^+^ T cell responses found after treatment with agonistic CD40 mAbs. Thus, our data suggest that agonistic CD40 mAb is an important adjuvant and acts to enhance and prolong immunity induced when using VSV-based vaccines.

## Materials and Methods

### Ethics statement

This study was carried out in strict accordance with the recommendations in the Guide for the Care and Use of Laboratory Animals of the National Institutes of Health. The animal experimental protocol (Protocol #2010–26 and 2013–25) was approved by the Institutional Animal Care and Use Committee (IACUC) at Montana State University (Federal-Wide Assurance Number: A3637-01).

### Mice

Female C57BL/6 mice between 5–8 weeks old were purchased from the Animal Resource Center at Montana State University. BAC transgenic Blimp-1 YFP reporter bone marrow was obtained from E. John Wherry (University of Pennsylvania). Blimp-1 YFP chimeras were generated by reconstituting C57BL/6 mice lethally irradiated (1000 rads) with 2×10^6^ Blimp-1 YFP bone marrow cells. Chimeric mice were rested 8 weeks before use.

### Pathogens, infections, and treatments

Both the recombinant VSV expressing ovalbumin [30] and recombinant *Listeria monocytogenes* (LM) expressing ovalbumin [24] have been previously described elsewhere. In all experiments, for primary infections mice were infected i.v. with either 2×10^5^ plaque-forming units (PFU) of VSV-ova or 2×10^3^ colony-forming units (CFU) of LM-ova. For protective immunity experiments, age-matched mice were challenged with 5×10^5^ CFU of LM-ova. To assess the functionality of the recall CD8^+^ T cells, age-matched mice were challenged with 5×10^4^ CFU of LM-ova.

To examine the role of PD-1 in regulating protective immunity at the time of pathogen re-challenge, vaccinated mice were treated with 300 µg of the blocking monoclonal antibody RMP1-14 [31]. To examine if overt stimulation of CD40 could enhance CD8^+^ T cell mediated protection, mice were treated with 100 µg of FGK4.5, an agonistic anti-CD40 monoclonal antibody (Bio-X-Cell), one day after infection. In other experiments FGK4.5 treated animals were further treated on days −1, +1, and +3 relative to infection with 300 µg of blocking monoclonal antibodies to CD70 (FR70), OX40L (RM134L), or ICOS-L (HK5.3) [32].

### Assessment of bacterial burden

The presence of *L. monocytogenes* in the spleen and liver was evaluated by dissociation of tissues through a 40-µm or 70- µm filter, respectively. Cells were collected by centrifugation at 2400 rpm for 15 minutes. Cells were suspended in 1% saponin (Acros) to release all bacteria. Serial dilutions of each tissue were plated onto brain–heart infusion agar plates containing 5 µg/ml erythromycin, and the numbers of colonies were enumerated 36 hours later.

### Tissue sample preparation and flow cytometric analysis

Cells were isolated from the spleen by digestion with 100 units/ml of collagenase (Gibco) at 37°C for 30 minutes, followed by disruption through a 40-µm filter. After which, red blood cells were lysed using a Tris ammonium chloride solution. Staining of ∼10^7^ cells was performed in 200 µl of PBS containing 2% bovine serum and 2 mM EDTA. For T cell analysis, Ova/K^b^-specific T cells were identified by staining with APC-labeled H-2K^b^ tetramer containing the ovalbumin derived peptide SIINFEKL, which was generated as previously described [33]. Tetramer staining was always conducted in the presence of anti-CD8a (clone 53.6.7). Staining with Ova/K^b^ tetramers and the appropriate antibodies for cell surface antigens was conducted at room temperature for one hour. Phenotypic analysis of T cells was conducted using a panel of cell surface markers: CD8a, CD3e, KLRG1, CD127, CD11a, and CD62L. Intracellular staining for the transcription factors T-bet, Eomes and Blimp1 (by anti-GFP antibody) was done after the tetramer/surface stain after fixation and permeabilization, as previously described [34]. For analysis of antigen-presenting cells, primary antibody staining and streptavidin-FITC labeling were both conducted at 4°C for 30 minutes each. Phenotypic analysis of antigen-presenting cells was conducted using a panel of cell surface markers: CD11b, CD11c, Ly6g, Ly6c, CD19, NK1.1, CD3e, I-A/I-E, CD8a, CD30L, ICOSL, CD80, CD86, CD40, CD70, or OX40L. All antibodies used for phenotypic analysis were purchased from either Biolegend, except CD11a which was from BD Biosciences and CD62L, CD11c, and CD30L which were from eBioscience. After staining cells were washed and fixed with 1% paraformaldehyde in PBS. Fluorescent intensities were measured using an LSR (BD Biosciences) and data were analyzed using FlowJo software (Tree Star). Fluorescent intensity of the co-stimulatory molecules on the dendritic cell population showed a normal distribution, which allows for the geometric mean fluorescent intensity to be used to compare expression levels.

### Intracellular cytokine staining

Spleens were harvested, crushed through a 40 µm filter, and cells were incubated with 1 or 0.001 µg/ml of the SIINFEKL peptide plus 1 µl GolgiPlug (BD Biosciences) in medium at 37°C for 4.5 h. Cells were stained for cell surface antigens on ice for 15 minutes. Cells were then fixed and rendered permeable using BD Cytofix/Cytoperm (BD Biosciences) before staining with antibodies to IFNγ, TNFα, and IL-2 (eBioscience). Fluorescent intensities were measured using an LSR (BD Biosciences) and data were analyzed using FlowJo software (Tree Star). Fluorescent intensity of IFNγ in the antigen-specific CD8^+^ T cell population showed a normal distribution, which allows for the geometric mean fluorescent intensity to be used to compare expression levels.

### Statistical analysis

Statistical significance was determined by either an unpaired Student’s t-test or one-way ANOVA with a Bonferroni’s post-test using Prism 5 (Graphpad Software). Bacterial burden was log10 transformed prior to statistical analysis, as previously discussed [35]. Significance was set as any p-value less than 0.05 (p<0.05).

## Results

### VSV infection induces short-lived CD8^+^ T cell mediated protective immunity

**Figure 1:**
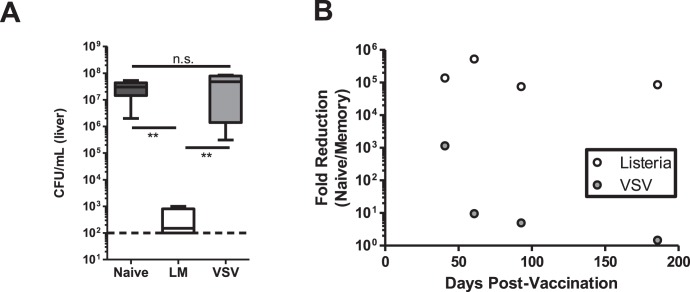
Protective immunity induced by VSV is short lived. C57BL/6 mice were i.v infected with 2×10^5^ pfu VSV-ova or 2×10^3^ cfu LM-ova. (A) 180 days after primary infection mice were re-challenged with 5×10^5^ cfu LM-ova. Three days later livers and spleens (data not shown) were plated to assess bacterial burden. Statistical significance was determined using a one-way ANOVA with a Bonferroni’s post-test (**p<0.01). (B) Naïve C57BL/6 or memory mice infected 30, 60, 90, or 180 days previously with either VSV-ova or LM-ova were challenged with 5×10^5^ CFU of LM-ova. Three days post-challenge bacterial burden was quantified in the spleen and liver. The fold reduction of LM-ova burden at 72 h post-challenge in vaccinated versus naïve animals was calculated from representative time-point out to ∼6 months post-vaccination. This was done by dividing the LM burden in the naïve animals by those found in vaccination animals to get a fold-reduction in LM burden. Data are representative of at least two independent experiments at each time-point.

Our previous data indicated that infection with either VSV-ova or LM-ova could induce robust memory CD8^+^ T cell responses capable of significant re-expansion, but protective immunity was not measured [14]. Recent work has demonstrated that CD8^+^ T cells induced by lymphocytic choriomeningitis virus (LCMV) or Influenza A virus (IAV) had different protective capacities [36]. In the present study, we wanted to similarly examine the protective ability of memory CD8^+^ T cells induced by either LM or VSV. To conduct this experiment, naïve C57BL/6 or “memory” mice that were previously infected (>4 months) with either VSV-ova or LM-ova were challenged with 5×10^5^ CFU of LM-ova. Three days later, we quantified bacterial burden in the spleen and liver. As expected mice previously infected with LM-ova were afforded nearly sterilizing protective immunity 72 h post-challenge [37], but surprisingly mice infected with VSV-ova >4 months prior were afforded minimal protective immunity (Figure 1a) even though their secondary expansion capability is similar [14]. Because VSV has previously been successfully used as a vaccine vector [38], [39], we next analyzed the protective immunity afforded by VSV-ova in a kinetic manner. To conduct these experiments naïve C57BL/6 or memory mice infected 30, 60, 90, or 180 days previously with either VSV-ova or LM-ova, as a positive control, were challenged with 5×10^5^ CFU of LM-ova. Three days post-challenge, we again quantified bacterial burden in the spleen and liver. While the protective immunity afforded by prior LM-ova infection was quite robust and stable at ∼100,000 fold reduction of bacterial burden, protective immunity afforded by prior VSV-ova infection rapidly waned (Figure 1b). Specifically, 30 days after VSV-ova infection those mice were protected ∼1,000-fold over control mice, which is still less immunity than afforded by prior LM-ova infection (Figure 1b). Interestingly, by 60 days after VSV-ova infection protective immunity waned to less than 10-fold over controls and continued to decay with time (Figure 1b). These data demonstrate that the memory population induced by VSV vaccination induces a memory CD8^+^ T cell population that only provides short-term protective immunity and, thus, is likely not programmed appropriately during initial T cell priming.

### VSV infection induces an alternative memory CD8^+^ T cell phenotype

**Figure 2:**
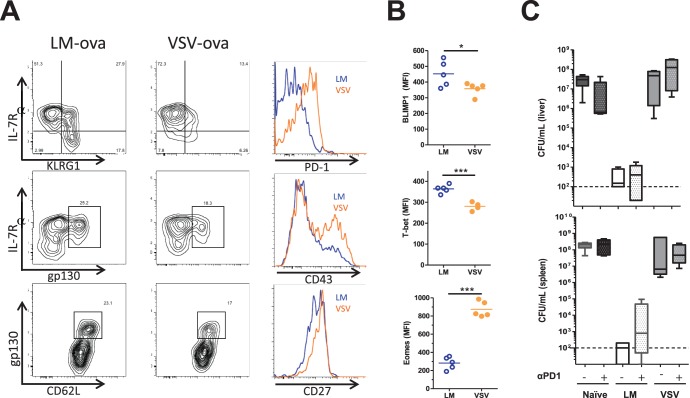
Memory CD8^+^ T cell induced by VSV and LM infection differ in their differentiation status. C57BL/6 mice were i.v infected with 2×10^5^ pfu VSV-ova or 2×10^3^ cfu LM-ova. Sixty days post-infection the cell surface phenotype (A) and transcription factor profile (B) of the Ova/K^b^-specific CD8^+^ T cell memory populations was assessed by flow cytometry. (A–B) Analysis is on gated specifically on CD11a^high^ Ova/K^b^-specific CD8^+^ T cells and the mean fluorescence intensity (MFI) of each transcription factors BLIMP1, T-bet, and Eomes from individual mice was graphed. Blue line represents LM-ova and orange line represents VSV-ova. (C) To test whether blocking the PD-1 receptor could induce protective immunity mice were treated with 300 µg of RMP1-14, a blocking mAb to PD-1, at the time of re-challenge. Mice were re-challenged with 5×10^5^ cfu LM-ova and three days later the bacterial burden in the liver and spleen was assessed. Statistical significance was determined using an unpaired Student’s t-test (*p<0.05; ***p<0.001). Data are representative of at least two independent experiments.

Past work has elegantly demonstrated that persistent high levels of antigen results in dramatic functional changes to CD8^+^ T cell populations [20], [40], but lower levels of persistent infection result in less impairment of the T cell response [41], [42], [43]. Specifically, the PD-1 receptor seems to be central in this functional impairment during chronic infections [44]. Interestingly, antigen can be detected for >30 days after inoculation during VSV infection [45], but the outcome with respect to CD8^+^ T cell-mediated immunity in the presence of persistent antigen during VSV infection of mice remains elusive. Thus, C57BL/6 mice were infected with either *L. monocytogenes* or VSV-ova or LM-ova, as a positive control, and the phenotype of the resultant splenic memory CD8^+^ T cell populations were analyzed 60 day after infection. Similar to our previous results [14], [46], the VSV memory CD8^+^ T cell population more quickly equilibrated to a CD27^high^ CD127^high^ KLRG1^low^ phenotype and contained a lower proportion of central-memory CD8^+^ T cells than *L. monocytogenes*-induced CD8^+^ T cells (Figure 2a). Interestingly, memory CD8^+^ T cells induced by either *L. monocytogenes* or VSV all the CD8^+^ T cells that expressed CD62L also expressed IL-6st/gp130 (Figure 2a). Memory CD8^+^ T cells induced by VSV expressed high levels of PD-1, while those induced by *L. monocytogenes* infection did not express PD-1 at these memory times (Figure 2a). Additionally, a proportion of the memory CD8^+^ T cells induced by VSV maintained expression of CD43 (1B11) while those induced by *L. monocytogenes* infection expressed minimal levels of CD43 (1B11) (Figure 2a).

Memory CD8^+^ T cells induced by VSV infection had an altered transcription factor profile expression compared to those induced by *L. monocytogenes*, expressing extremely high levels of Eomes and slightly lower levels of both T-bet and BLIMP1 (Figure 2b). Because the memory CD8^+^ T cells induced by VSV expressed cell surface markers (PD-1) and transcription factors (Eomes) associated with functional exhaustion [34], [44], we wanted to test whether inhibition of PD-1 released the VSV-induced memory CD8^+^ T cell population allowing it to provide robust protection immunity. To test this hypothesis C57BL/6 mice were either left unvaccinated, vaccinated with LM-ova, as a positive control, or vaccinated with VSV-ova. Ninety days later mice were treated with 300 µg of either an isotype control mAb or a blocking anti-PD1 mAb (RMP1-14) one day prior and one day after challenge with 5×10^5^ CFU of LM-ova. Three days later we quantified bacterial burden in the spleen and liver. Similar to our earlier results, the protective immunity afforded by prior LM-ova infection was quite robust, while minimal protective immunity was afforded by prior VSV-ova infection (Figure 2c). Treatment of mice with a blocking anti-PD1 mAb (RMP1-14) during secondary challenge did not allow VSV-induced memory Ova/K^b^-specific CD8^+^ T cells to afford protective immunity. Thus, our data demonstrate that while VSV-induced memory CD8^+^ T cells have a phenotype which partially resembles functionally exhausted CD8^+^ T cell; however, blocking PD-1 did not allow those cells to provide protective functions.

### Agonistic anti-CD40 enhances VSV-induced CD8^+^ T cell response

**Figure 3:**
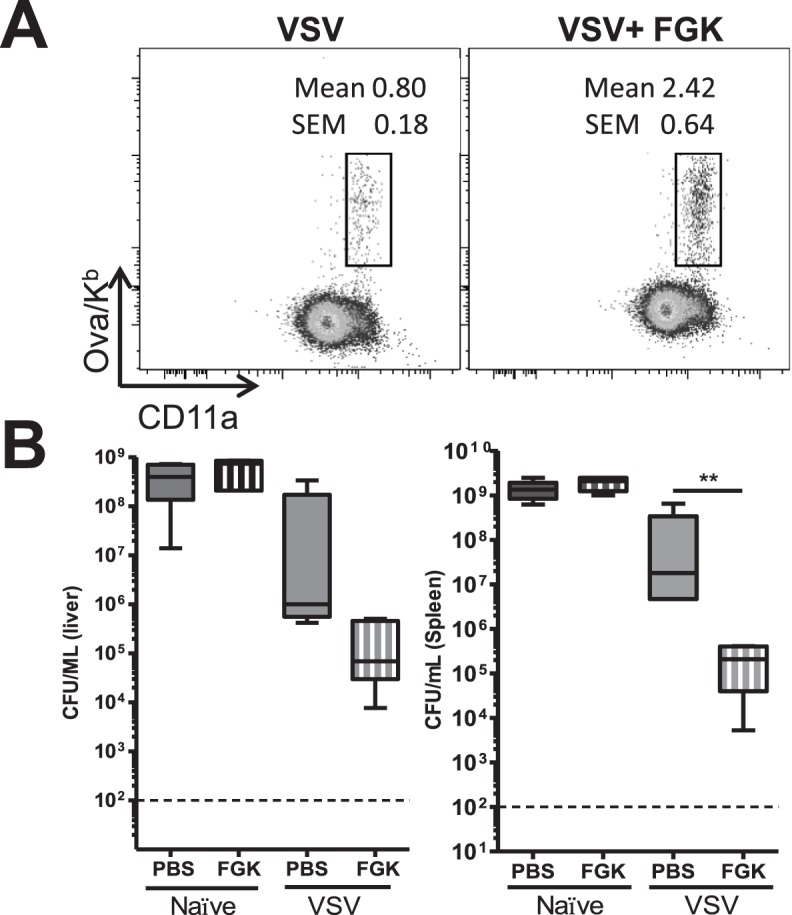
FGK4.5 treatment enhances CD8^+^ T cell responses induced by VSV infection. C57BL/6 mice were i.v. infected with 2×10^5^ pfu VSV-ova and treated i.p. with 100 µg FGK4.5, an agonistic CD40 mAb, one day post infection. (A) Sixty days after primary infection the Ova/K^b^-specific CD8^+^ memory T cell population in the blood was quantified by Ova/K^b^-tetramer staining. (B) To assess whether activating CD40 would induce protective immunity, vaccinated mice were re-challenged with 5×10^5^ cfu LM-ova sixty day after VSV-ova vaccination. Three days later spleens and liver were assessed for bacterial burden. Statistical significance was determined using a one-way ANOVA with a Bonferroni’s post-test (**p<0.01). Data are representative of at least three independent experiments.

As our results demonstrate VSV-induced protective T cell immunity was short-lived (Figure 1b) and had an alternative memory phenotype (Figure 2A–B), suggesting that the CD8^+^ T cell response may not be appropriately programed during priming. CD4^+^ T cell help is thought to be important for the generation and maintenance of memory CD8^+^ T cell populations [47], [48]. Additionally, the CD40–CD154 co-stimulatory pathway can mediate CD4^+^ T cell help [23]. Interestingly, induction of a robust CD8^+^ T cell response against *L. monocytogenes* is dependent on CD4^+^ T cell help and CD40 signaling; while, during VSV infection the induction of a strong CD8^+^ T cell response occurs even in the absence of CD4^+^ T cells and CD40 signaling [25], [29]. Here we wanted to test whether the provision of overt CD40 signaling using the agonistic anti-CD40 mAb (FGK4.5) would enhance the protective immunity of memory CD8^+^ T cells induced during VSV infection. To test this hypothesis C57BL/6 mice were either left unvaccinated or vaccinated with VSV-ova. One day later mice were treated with 100 µg of either an agonistic anti-CD40 mAb (FGK4.5) or PBS, treatment with an isotope control mAb gave analogous results (data not shown). Sixty days later the magnitude of the Ova/K^b^-specific memory CD8^+^ T cell population in the peripheral blood was quantified by tetramer staining. Similar to our previous studies [14], VSV infection resulted in the generation of a robust memory CD8^+^ T cell population (Figure 3a), which was larger than that induced by LM-ova infection (data not shown and [14]). Importantly, treatment of VSV infected mice with 100 µg of agonistic anti-CD40 mAb (FGK4.5) one day after infection resulted in a nearly three-fold larger memory CD8^+^ T cell population (Figure 3a). Next we wanted to assess whether those memory CD8^+^ T cells could now afford protective immunity against LM-ova infection; thus, mice were challenged with 5×10^5^ CFU of LM-ova and three days later bacterial burden in the spleen and liver was quantified. Similar to our earlier results the protective immunity afforded by prior VSV-ova infection was minimal in both the spleen and liver (Figure 3b). Treatment of mice with the agonistic anti-CD40 mAb (FGK4.5) during priming of the CD8^+^ T cells during primary VSV infected generated a memory CD8^+^ T cell population that was protective against LM-ova challenge. Thus, our data demonstrate that provision of CD40 signaling could enhance the size of the VSV-induced memory CD8^+^ T cell population and allow it to provide protective immunity against subsequent LM-ova infection.

### Agonistic anti-CD40 enhances the functionality of secondary effector CD8^+^ T cells

**Figure 4:**
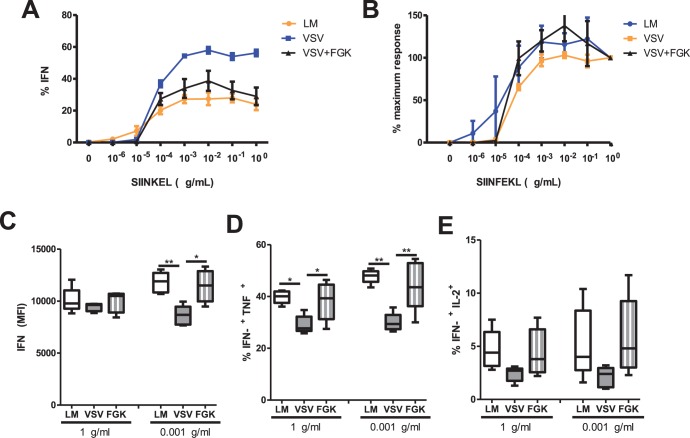
FGK4.5 treatment during VSV vaccination enhances the functional avidity and poly-functionality of secondary effector CD8^+^ T cells. C57BL/6 mice were i.v. infected with 2×10^3^ cfu LM-ova, or with 2×10^5^ pfu VSV-ova, or 2×10^5^ pfu VSV-ova plus treated i.p. with 100 µg FGK4.5 one day post infection. Ninety days after primary challenge mice were re-challenged with 5×10^4^ cfu LM-ova. Seven days later the sensitivity to antigen and functionality of the secondary effector CD8^+^ T cell response was assessed by intracellular cytokine staining. Spleen lymphocytes were stimulated with one to 0.00001 µg/mL of the SIINFEKL peptide. Cells were fixed and rendered permeable before staining with antibodies IFNγ, TNFα, and IL-2. We tested (A) the percent IFNγ and (B) the percent IFNγ over the maximum; at all peptide dilutions. Next we analyzed the (C) geometric mean fluorescence intensity (MFI) of the CD8^+^ T-cells for IFNγ, the (B) percent of IFNy expressing cells also expressing TNFα and (E) the IFNγ expressing cells also expressing IL-2. Results shown for 0.001 and 1 µg/mL SIINFEKL only (Figures C, D, E). Statistical significance was determined using a one-way ANOVA with a Bonferroni’s post-test ***p<0.001**p<0.01; *p<0.05). Each group contained 4–5 mice per group.

As our results demonstrate the VSV-induced CD8^+^ T cell response was enhanced in magnitude and protective ability following agonistic anti-CD40 mAb (FGK4.5) treatment (Figure 3). We also wanted to test whether treatment with agonistic anti-CD40 mAb (FGK4.5) enhanced the sensitivity of the CD8^+^ T cells to antigen and/or their polyfunctionality. We previously showed that VSV infection induced CD8^+^ T cells with decreased polyfunctionality [14]. To test the hypothesis that enhancing CD40 signaling would enhance the polyfunctionality of CD8^+^ T cells after VSV vaccination, C57BL/6 mice were vaccinated with LM-ova (as a positive control), VSV-ova, or VSV-ova followed one day later with 100 µg of agonistic anti-CD40 mAb (FGK4.5). Ninety days post-infection the mice were challenged with 5×10^4^ CFU of LM-ova. Seven days later the sensitivity to antigen and polyfunctionality of the responding secondary effector CD8^+^ T cells was assessed by intracellular cytokine staining for IFNγ, TNFα, and IL-2. The dose response curves of the Ova-specific memory CD8^+^ T cell from the three experimental groups did not differ when the percent IFNγ^+^ cells was analyzed (Figure 4a–b). Interestingly, at saturating levels of SIINFEKL peptide (1 µg/ml) secondary effector CD8^+^ T cells from mice originally vaccinated with LM-ova, VSV-ova, and VSV-ova plus FGK4.5 produced equivalent amounts of IFNγ on a per cell basis (Figure 4c), but there was a significantly decreased proportion of IFNγ^+^ TNFα^+^ dual producing CD8^+^ T cells in VSV vaccinated mice, which were enhanced by treatment with agonistic anti-CD40 (Figure 4d). Furthermore, after VSV vaccination there was a trend toward fewer IFNγ^+^ IL-2^+^ dual producing CD8^+^ T cells, which were enhanced by treatment with agonistic anti-CD40 mAb (FGK4.5) (Figure 4e). Interestingly, at sub-optimal dose of SIINFEKL peptide (0.001 µg/ml) the same trend in polyfunctional CD8^+^ T cells was observed (Figure 4d and 4e), but there was also a defect in IFNγ production on a per cell basis, which could be rescued by treatment with agonistic anti-CD40 (Figure 4c). Thus, our data demonstrate that provision of CD40 signaling enhances the functionality of CD8^+^ T cells induced by VSV infection, especially at sub-optimal levels of antigen which may be critical for the early detection and clearance of a pathogen before it become established in the host.

### Agonistic anti-CD40 enhances dendritic cell maturation during VSV infection

**Figure 5:**
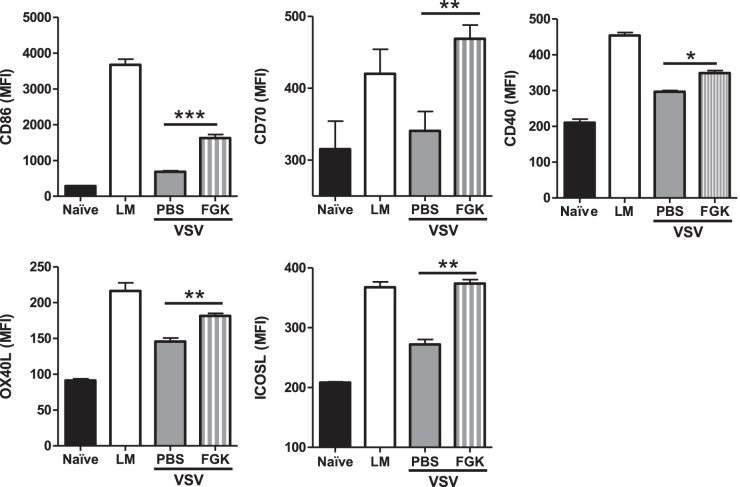
FGK4.5 treatment enhances dendritic cell maturation during VSV infection. C57BL/6 mice were either left uninfected or challenged with 2×10^3^ cfu LM-ova, or 2×10^5^ pfu VSV-ova with one group of VSV-ova infected mice treated i.p. with 100 µg FGK4.5 one day after infection. Three days after infection expression of the co-stimulatory molecules CD86, CD70, CD40, OX40L, and ICOSL on splenic dendritic cells (B220^neg^ CD3^neg^ NK1.1^neg^ F4/80^neg^ CD11c^high^ I-A/I-E^high^) was assessed. Data are representative of two independent experiments, with each experimental group containing 4–5 mice. Graphs represent the geometric mean fluorescent intensity (MFI) of each co-stimulatory marker ± one standard error. Statistical significance was determined using an one-way ANOVA with a Bonferroni’s post-test (***p<0.001; **p<0.01).

We next wanted to examine how agonistic anti-CD40 treatment would enhance the CD8^+^ T cell response during VSV infection. The matrix protein of VSV is known to have an immunosuppressive effect and can specifically impair the maturation of dendritic cells during VSV infection [49]. Using a subunit vaccination protocol, it has been shown that the synergy observed between TLR and agonistic anti-CD40 mAb as adjuvant reflects their ability to activate dendritic cells [50]. VSV infection will activate TLRs [51], [52], thus we wanted to examine whether provision of agonistic anti-CD40 would enhance the activation of dendritic cells. To test this hypothesis C57BL/6 mice were left uninfected, infected with LM-ova (as a positive control), infected with VSV-ova, or infected with VSV-ova and treated one day later with 100 µg of agonistic anti-CD40 mAb (FGK4.5). Three days after infection, dendritic cell maturation in the spleen was assessed. Dendritic cells were identified as CD3^−^ NK1.1^−^ CD19^−^ Ly6g^−^ CD11c^high^ MHC-II^high^. Splenic dendritic cells from naïve C57BL/6 mice expressed minimal levels of all co-stimulatory molecules examined. Further, we found that LM infection induced high levels of CD86, CD70, CD40, OX40L, and ICOS-L on dendritic cells, while VSV infected mice did not induce CD86 or CD70 and induced very low levels of CD40, OX40L and ICOS-L (Figure 5). Treatment of VSV infected animals with a single dose of FGK4.5 significantly enhanced the expression of co-stimulatory molecules on dendritic cells, including CD86, CD70, OX40L, and ICOS-L (Figure 5). Thus, provision of CD40 signaling can enhance dendritic cells maturation during VSV infection.

### Agonistic anti-CD40 enhancement of the VSV-induced CD8^+^ T cell response occurs through a CD70-dependent mechanism

**Figure 6:**
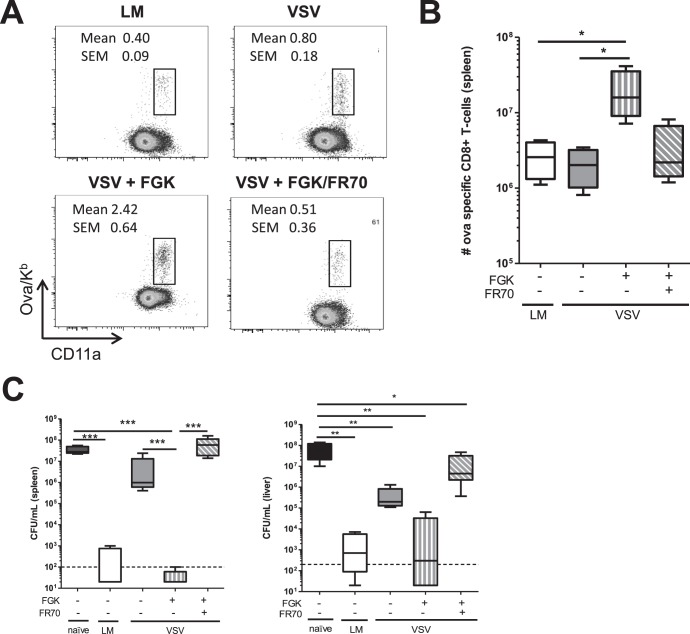
Enhancement of CD8^+^ T cell response by FGK4.5 treatment during VSV infection is dependent on CD70. C57BL/6 mice were left uninfected, or i.v. infected with 2×10^3^ cfu LM-ova, or with 2×10^5^ pfu VSV-ova alone, or 2×10^5^ pfu plus 100 µg FGK4.5 treated i.p. one day after infection, or 2×10^5^ pfu VSV-ova plus 100 µg FGK4.5 one day post infection and 300 µg FR70, a blocking CD70 mAb, on days −1, +1, and +3 relative to infection. The magnitude of the Ova/K^b^-specific memory CD8^+^ T cell population in the (A) peripheral blood and in the (B) spleen was quantified 60 days post infection. (C) To assess protective immunity, memory mice were re-challenged with 5×10^5^ cfu LM-ova. Three days later bacterial burden was assessed in the spleen and liver. Each experiment contained 4–5 mice per group and data are representative of at least two independent experiments. Statistical significance was determined using a one-way ANOVA with a Bonferroni’s post-test (***p<0.001; **p<0.01; *p<0.05).

As our results demonstrate, treatment with an agonistic anti-CD40 mAb (FGK4.5) during VSV infection resulted in maturation of dendritic cells (Figure 5). We next wanted to explore whether any of the co-stimulatory molecules increased by agonistic anti-CD40 mAb (FGK4.5) treatment where responsible for enhancing the magnitude and protective abilities of the memory CD8^+^ T cell populations. To test whether these co-stimulatory molecules were crucial for enhancing memory formation and protective immunity after agonistic anti-CD40 mAb (FGK4.5) treatment during VSV infection, C57BL/6 mice were vaccinated with VSV-ova, VSV-ova followed by treatment with 100 µg of agonistic anti-CD40 mAb (FGK4.5) one day after VSV administration, or VSV-ova followed by treatment with 100 µg of an agonistic anti-CD40 mAb (FGK4.5) one day after VSV administration in the presence of 300 µg of a blocking mAbs to CD70 (FR70) administered −1, +1, and +3 d relevant to VSV infection. Additionally, groups of unvaccinated or LM-ova vaccinated mice were used as negative and positive controls, respectively. Sixty days later the magnitude of the Ova/K^b^-specific memory CD8^+^ T cell population in the peripheral blood was quantified by tetramer staining. VSV infection resulted in the generation of a robust memory CD8^+^ T cell population, which was larger than that induced by LM-ova infection (Figure 6a–b). Similar to our earlier results, treatment of VSV-ova infected mice with 100 µg of agonistic anti-CD40 mAb (FGK4.5) one day after infection resulted in a substantially larger memory CD8^+^ T cell population (Figure 6a–b). Blockade of CD70 during vaccination with VSV-ova plus agonistic anti-CD40 mAb (FGK4.5) treatment reduced the memory CD8^+^ T cell population back to levels observed with VSV-ova alone (Figure 6a–b). Next we wanted to assess whether the memory CD8^+^ T cells could afford protective immunity against LM-ova infection; thus, mice were challenged with 5×10^5^ CFU of LM-ova and three days later bacterial burden in the spleen and liver was quantified. Similar to our earlier results the protective immunity afforded by LM-ova vaccination was robust, while protective immunity afforded by VSV-ova vaccination was minimal (Figure 6c). Treatment of mice with the agonistic anti-CD40 mAb (FGK4.5) during priming of the CD8^+^ T cells during primary VSV infected generated a memory CD8^+^ T cell population that was protective against LM-ova challenge, which was completely abrogated by blockade of CD70 during vaccination (Figure 6c). Thus, our data demonstrate that provision of CD40 signaling enhances the size of the VSV-induced memory CD8^+^ T cell population and allow it to provide protective immunity against subsequent LM-ova infection through a CD70-dependent pathway.

## Discussion

The development of T cell-inducing vaccines is highly sought after for prophylactic and therapeutic interventions against intracellular infections, chronic infections, and cancers. Because of this, extensive numbers of studies have examined the developmental cues that regulate the differentiation of effector and memory CD8^+^ T cells. One important observation has been that different infections and vaccination regimens favor different effector and memory differentiation pathways [13], [14]. Thus, we wanted to examine the protective immunity afforded by memory CD8^+^ T cells induced by VSV vaccination. Here, we have shown that while VSV infection induces a sizable memory CD8^+^ T cell population, it only conferred a transient period of protective immunity. Importantly, memory CD8^+^ T cell induced by VSV displayed a phenotype of partially dysfunctional T cells, expressing high levels of PD-1 and having a reduced proportion of IFNγ^+^ TNFα^+^ polyfunctional CD8^+^ T cells. Provision of CD40 signaling during CD8^+^ T cell priming in VSV infections resulted in enhanced CD8^+^ T cell responses, which was dependent on CD27:CD70 signaling.

While the induction of sufficiently sized memory CD8^+^ T cell populations is necessary for providing protective immunity [53], another important consideration is the functionality of those cells [18], [19]. Our data demonstrate that while both VSV and LM can be used as effective vaccine vectors for inducing protective memory CD8^+^ T cell over the first 1–2 months, the protective quality of VSV-induced memory CD8^+^ T cells waned over time (Figure 1) while the magnitude of the memory CD8^+^ T cell population remained stable over time [14]. Thus, long-term quality of the memory CD8^+^ T cells needs to be considered when assessing new vaccination modalities. This is especially true for VSV which has been proposed as a good vaccine vector for the induction of CD8^+^ T cells [17].

In HCMV infected individuals, CMV-specific antibody levels directly correlated with increased morbidity and mortality risk in elderly individuals [54], while the TCRαβ diversity in CD8^+^ T cells was inversely correlated with anti-CMV IgG antibody titers [55]. Thus, it appears that a high diversity of TCRαβ among the CD8^+^ T cells may be an important factor in limiting CMV-associated morbidity and mortality [55]. Other studies demonstrated that highly diverse TCRαβ usage enhances the selection of high-avidity CD8^+^ T cells [56], [57]. Thomas and colleagues postulated that either CMV-specific antibodies limit the TCRαβ by clearing viral antigens or chronic antigen exposure may lead to a narrowing of the TCRαβ repertoire [55]. It is possible that an analogous situation occurs during VSV infection. VSV infection results in a robust early antibody response [58], which could alter the context or magnitude of viral antigen presentation to CD8^+^ T cells. Also, VSV RNA and antigens can persist long-term in infected mice [45], [59], [60], which could alter the repertoire and/or function of the CD8^+^ T cells. VSV infection resulted in an altered cell surface phenotype and transcription factor expression profile (Figure 2), protective function (Figure 1), and cytokine profile (Figure 4) of the memory CD8^+^ T cell populations in a manner which has some similarities to what is observed during chronic viral infections [61]. However, blockade of the inhibitory receptor PD-1 at the time of challenge did not improve the protective capacity of VSV-induced CD8^+^ T cells (Figure 2). Thus, further studies are needed to dissect the effects of chronic antigenic stimulation to CD8^+^ T cells after VSV infection.

It is possible that the rapid control of VSV by natural antibodies may actually impair the maturation of dendritic cells or other antigen-presenting cells. Indeed, we found that after VSV infection dendritic cell maturation was substantially blunted (Figure 5). One pathway necessary for activation of dendritic cells is CD4^+^ T cell help and/or CD40:CD154 signaling [23], [26]. Interestingly, induction of CD8^+^ T cell responses by LM is known to be highly dependent on CD4^+^ T cell help and CD40 signaling, while the CD8^+^ T cell response induced by VSV occurred in the absence of CD4^+^ T cells and CD40 [25], [29]. Thus, we tested whether the provision of CD40 signaling using an agonistic mAb could enhance dendritic cell maturation and, subsequently, the quality of the CD8^+^ T cell response. Interestingly, provision of CD40 signaling resulted in enhanced dendritic cell maturation as demonstrated by elevated levels of CD86, CD70, CD40, OX40L, and ICOS-L on dendritic cells (Figure 5). This finding is strikingly similar to what Kedl and colleagues have found during subunit vaccination using peptides plus both a TLR agonist and agonistic anti-CD40 mAb [50]. In their vaccination protocol, the action of the agonistic anti-CD40 mAb was highly dependent on the induction of CD70 [27], [50] and to a lesser extent OX40L [62]. Coinciding with this finding, we demonstrate that enhancement of the VSV-induced CD8^+^ T cell response after agonistic anti-CD40 mAb treatment was highly dependent on CD70 (Figure 6).

Our results demonstrate that while the induction of sufficiently sized memory CD8^+^ T cell populations is necessary for providing protective immunity, it is equally important to consider the functionality and protective longevity of those cells. We showed that cytokine production by VSV-induced CD8^+^ T cells was impaired during recall infection (Figure 4), even though we previously showed that VSV-induced memory CD8^+^ T cells were able to undergo robust secondary expansion after LM-ova infection [14]. Mueller *et al* demonstrated that LCMV-induced memory CD8^+^ T cells were more protective than IAV-induced memory CD8^+^ T cells, which correlated with enhanced cytokine production and proliferation by LCMV-induced memory CD8^+^ T cells upon recall [36]. This finding is intriguing because, similar to VSV, IAV-derived antigens were able persist for >60 days post-infection [63] and both IAV and VSV infection induce robust CD8^+^ T cell responses in the absence of CD4^+^ T cell-mediated help [25], [29], [64]. Our findings, together with those of Mueller *et al* [36], point to the critical importance of assessing the protective quality of vaccine-induced memory CD8^+^ T cells population, or to develop better correlates of protective immunity, than simply quantifying the magnitude of the memory CD8^+^ T cell population.

## Conclusions

Our results demonstrate that VSV vaccination is impaired in its ability to induce highly protective memory CD8^+^ T cells. CD40 signaling is likely to be crucial for the generation of high quality high CD8^+^ T cells populations, which is mediated through CD27:CD70 signaling. This information is crucially important for the development of T cell vaccines, which must work in the absence of effective CD4^+^ T cells populations, such as during HIV infection.
